# Oral artemisinin monotherapy removal from the private sector in Eastern Myanmar between 2012 and 2014

**DOI:** 10.1186/s12936-016-1292-8

**Published:** 2016-05-23

**Authors:** Hnin Su Su Khin, Tin Aung, Aung Thi, Chris White, ACTwatch Group

**Affiliations:** Population Services International Myanmar, No. 16, Shwe Gon Taing Street 4, Yangon, Myanmar; National Malaria Control Program, Department of Public Health, Ministry of Health, Naypyidaw, Myanmar; Division of Global Policy & Advocacy, Bill & Melinda Gates Foundation, Seattle, WA USA; Population Services International, 1120 19th St NW Suite 600, Washington, DC 20036 USA

**Keywords:** Antimalarial drug resistance, Malaria elimination, Outlet survey, Oral artemisinin monotherapy, Artemisinin combination therapy, Subsidy

## Abstract

**Background:**

In 2012 the Artemisinin Monotherapy Therapy Replacement (AMTR) project was implemented in Eastern Myanmar to increase access to subsidized, quality-assured artemisinin combination therapy (ACT) and to remove oral artemisinin monotherapy (AMT) from the private sector. The aim of this paper is to examine changes over time in the private sector anti-malarial landscape and to illustrate the value of complementary interventions in the context of a national ACT subsidy.

**Methods:**

Three rounds of cross-sectional malaria medicine outlet surveys were conducted, in 2012, 2013 and 2014. Project intervention areas were selected from the Myanmar Artemisinin Resistance Containment (MARC) area. Provider detailing was implemented in these selected areas. Comparison areas were selected outside of this catchment area, from townships in close proximity to the MARC framework. Within each domain, multi-staged sampling was used to select areas for the survey. Outlets with the potential to sell or distribute anti-malarials in the private sector were screened for eligibility.

**Results:**

The total number of outlets approached for an interview was as follows in the intervention and comparison areas, respectively: 2012, N = 2046 and 1612; 2013, N = 1636 and 1884; 2014, N = 2939 and 2941. The percentage of pharmacies, general retailers and mobile providers (classed as ‘priority outlets’) with oral AMT in stock on the day of the survey decreased over time in the intervention areas (2012 = 68 %; 2013 = 48 %; 2014 = 10 %). Conversely, quality-assured ACT availability increased among these outlets (2012 = 4 %; 2013 = 62 %; 2014 = 79 %). Relative oral AMT market share among priority outlets also decreased over time (2012 = 44 %; 2013 = 18 %; 2014 = 14 %), while market share of quality-assured ACT increased (2012 = 3 %; 2013 = 59 %; 2014 = 51 %). Among priority outlets in the comparison area, similar trends were observed, though changes over time were less substantial compared to the intervention area. Other outlet types (community health workers and health facilities) performed relatively well over time though modest improvements were also observed.

**Conclusion:**

The findings point to the successful design and implementation of a strategy to rapidly remove oral AMT from pharmacies, general retailers and mobile providers and to replace its use with quality-assured ACT. The evidence also highlights the importance of supporting interventions in the context of a high-level subsidy.

**Electronic supplementary material:**

The online version of this article (doi:10.1186/s12936-016-1292-8) contains supplementary material, which is available to authorized users.

## Background

Continued use of oral artemisinin-based monotherapy is widely considered to be one of the main contributing factors to the development and spread of resistance to artemisinin and its derivatives [1]. Since 2006, the World Health Organization (WHO) urged countries and pharmaceutical companies to cease the marketing and use of oral artemisinin monotherapy in both the public and private sectors [2]. This was of acute concern given evidence of emerging artemisinin resistance in the Greater Mekong Sub-region (GMS), which was initially confirmed along the Cambodia–Thailand border but is now widespread in most of mainland Southeast Asia [3–7], including Myanmar [8, 9].

Myanmar is critical to the global containment of resistance; it has a far higher burden of disease than the rest of the GMS combined, low levels of investment in malaria control and health system strengthening [10, 11], extensive cross-border migration [12], hard-to-reach forested areas [13], and high consumption of incomplete doses of oral artemisinin monotherapy, namely from the private sector [11]. In 2011, the situation in Myanmar was particularly challenging given estimates that approximately 1.3 million adult equivalent treatment doses (AETD) of oral artemisinin monotherapy were being imported into Myanmar each year in the private sector, primarily artesunate tablets [14]. At the same time, substantial gains had been made in malaria control efforts, with reductions in malaria incidence rates attributed to increased intervention coverage [11]. For example, data from 135 townships found that between 2007 and 2011 the proportion of fever patients seeking treatment from village malaria workers testing positive for malaria decreased across all sites from an average of 41 to 24 %, respectively [11].

Myanmar’s efforts to ban the production, marketing authorization, export, import, and use of oral artemisinin monotherapy commenced in 2011 with a ban on the importation of artesunate and, in 2012, with a subsequent ban on the importation of all artemisinin-based monotherapy. However, oral artemisinin monotherapy could still be distributed and purchased in Myanmar while existing stocks were used up. There was also a 5-year window through which production and import licenses agreed upon prior to the ban would remain legal, increasing the likelihood that oral artemisinin uptake would continue in Myanmar for some time.

**Fig. 1:**
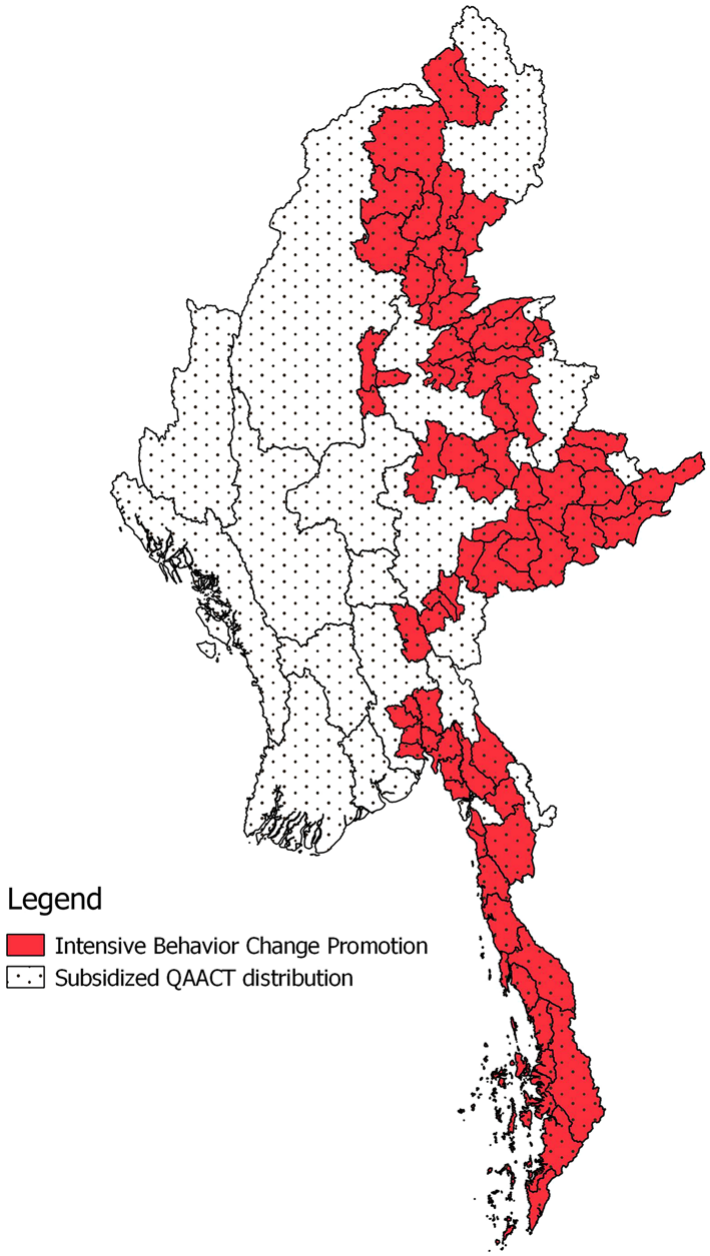
Map of AMTR project areas

Several steps have been taken by the country to help contain and prevent the spread of further artemisinin resistance. In 2011, the Myanmar Artemisinin Resistance Containment (MARC) project was implemented in the eastern part of the country to ensure improved access to the country’s first-line artemisinin-based combination therapy (ACT) treatment for uncomplicated falciparum malaria. While several activities were implemented by in-country partners and the National Malarial Control Program (NMCP), to address the private sector supply-side challenges, the Artemisinin Monotherapy Replacement Project (AMTR) was implemented by the international non-profit organization Population Services International (PSI)/Myanmar (Fig. 1). The AMTR project was designed to rapidly replace the widespread availability and use of oral artemisinin monotherapy with subsidized quality-assured ACT (ACT that comply with the Global Fund to Fight AIDS, Tuberculosis and Malaria’s Quality Assurance Policy). Efforts targeted distribution and sales in the private sector [15] given evidence that an estimated 50–80 % of people in Myanmar seek treatment from this sector [16].

The national scale of the AMTR project and its ability to shape the anti-malarial market in Myanmar could be a key asset in national efforts to delay artemisinin resistance and move toward the pre-elimination phase of malaria elimination in accordance with the GMS Malaria Elimination Plan [17]. Understanding the potential impact of the AMTR intervention will help to examine the importance of a high-level subsidy for quality-assured ACT as well as the role of other supporting interventions. The main aim of this paper is to show changes over time in the private sector anti-malarial landscape as a result of the AMTR project in eastern Myanmar between 2012, 2013 and 2014. A secondary objective is to illustrate the role of supportive provider interventions focused on priority outlets in targeted areas in eastern Myanmar as a means to illustrate the value of complementary interventions in the context of a national ACT subsidy and efforts to remove oral artemisinin monotherapy from the private sector market.

## Methods

The methodology for the project was adapted from the ACTwatch project [18]. The ACTwatch project is a multi-country research project implemented by PSI and launched in 2008. The goal of the ACTwatch project is to provide timely, relevant and high-quality anti-malarial market evidence to inform and monitor national and global policy, strategy and funding decisions for improving malaria case management. Since the project’s inception, standardized tools and approaches have been employed to provide comparable data across countries and over survey rounds. These approaches have been documented elsewhere in detail [18–22].

### Description of the interventions

A core component of the AMTR project intervention was the national distribution of subsidized quality-assured ACT [23]. Supply chain research identified the largest market-sharing company importing artesunate into Myanmar (AA Pharmacy), with 70 % of oral artemisinin monotherapy distributed by this importer [24]. Negotiations between PSI and AA Pharmacy led to an agreement to stop importing the product in late 2011 and to instead distribute subsidized, quality-assured ACT sold to end users at 500 Kyat (or around $0.75 in 2012). The quality-assured ACT (artemether–lumefantrine) was branded *Supa Arte*^®^ and over-packaged by PSI. The packaging included a quality-assurance lotus leaf logo for consumer recognition of the brand as a high-quality WHO and nationally recommended medicine.

The AMTR project also implemented a series of behavior change communication activities to promote the use of quality-assured ACT among consumers, using mass media activities. The overall objective of these activities was to increase demand for the national first-line ACT, which could be recognized by patients from the ‘quality seal’. ACT sales and mass media were further reinforced in the target area by intensive pharmaceutical detailing operations targeting priority outlet types through PSI product promoters as a means to amplify increases in ACT uptake in the supply chain. This supportive intervention specifically targeted three types of informal private providers in the intervention areas: pharmacies, general retailers, and mobile providers (termed ‘priority outlets’). These providers were chosen because the 2012 outlet survey showed that these types of providers were commonly stocking and distributing oral artemisinin monotherapy [23] and were generally underserved, with little or no ties to the government or non-governmental organizations (NGOs) for training, supervision or access to quality-assured commodities. The product promoters provided key messaging to providers as well as job aids and other awareness-raising tools regarding malaria case management. The product promoters did not sell ACT directly to the priority outlets but established links between the outlets and wholesalers supplying the quality-assured ACT. To ensure an affordable price for consumers, promoters emphasized a recommended retail price for the quality-assured ACT. Priority outlets were followed up with every 3 months by the PSI product promoters.

Priority outlets and non-priority outlets in both domains received distributions of subsidized ACT and were subject to the ban on oral artemisinin monotherapy as well as behavior change communication through mass media.

### Design and sampling

Three rounds of cross-sectional malaria medicine outlet surveys were conducted in eastern Myanmar, in 2012 (March–May), 2013 (August–October), and 2014 (September–October). Data collection coincided with the Myanmar rainy season when malaria transmission is highest, generally beginning in late June and ending in December [11].

Each survey was stratified to deliver separate estimates for two areas: the ‘intervention’ area, which received additional product promotion through medical detailing from PSI, and the ‘comparison’ area (areas for which this supportive intervention was not implemented). Intervention areas were defined as being located in the MARC project area and selected from this project area. The comparison area was defined as locations in close proximity to the Myanmar Artemisinin Resistance Containment (MARC) framework. There was no randomization of the intervention and comparison areas as the MARC area was pre-defined as a priority area for containment activities given artemisinin drug resistance risk.

The study was powered to detect a minimum of a 15 % point change in availability of quality-assured ACT between the two strata (intervention and comparison) at each survey round. Based on the desired number of anti-malarial stocking outlets within each round and assumptions about the number of anti-malarial stocking outlets per township, a sample of townships was selected within each research domain with probability proportional to population size, using a multi-staged cluster sampling approach. Within each selected urban ward or rural village tract (administrative boundaries in Myanmar), all private sector outlets with the potential to provide anti-malarials to patients were sampled (see Table 1 for a description of the outlet types). Further details of the sample size assumptions and sample selection are described elsewhere [25]. A census approach was used to identify outlets. Outlets were eligible for a provider interview and malaria product audit if they met at least one of the study criteria: (1) one or more anti-malarials reportedly in stock the day of the survey; and, (2) one or more anti-malarials reportedly in stock within the 3 months preceding the survey.

**Table 1:** Description of outlet types and classification

Priority outlets	
Pharmacies	Pharmacies are licensed by the Ministry of Health and are authorized to sell all classes of medicines including prescription-only medicines
General retailers	General retailers are grocery stores and village shops that sell fast-moving consumer goods, food and provisions. Although retailers may have over-the-counter medicines including anti-malarials available, national authorities do not regulate the sale of medicines by retailers
Mobile providers	Mobile providers selling medicines and other goods. They are not registered with any national regulatory authority

Non-priority outlets	
Private health facilities	Private general practitioners providing patient services within privately owned facilities that are licensed by the Ministry of Health. These practitioners may have formal or informal ties with government health facilities including serving on staff at government facilities and/or accessing government or non-government not-for-profit medicine supplies
Community health workers	Community-based health workers provide patient services and typically are linked with the government or NGOs, or other private health facilities and medical supply agents

### Training and fieldwork

For all survey rounds, interviewer training was provided over a minimum of six days using standardized training materials. The training focused on outlet identification, informed consent procedures and procedures for completing the questionnaire. An additional file shows the full outlet survey questionnaire (see Additional file 1). An additional two-day training was provided for quality controllers and supervisors.

Field workers were provided with a list of the selected administrative areas, maps that illustrated the administrative boundaries, and any existing official lists of facilities. Snowball sampling was used by field workers to identify facilities that were not on official lists. In each selected cluster, field workers conducted a census of all outlets that had the potential to provide anti-malarials by traveling systematically throughout the selected area. For each outlet that was identified during the census, a field worker then approached the outlet’s main provider or owner, invited him or her to participate in the study and screened for eligibility. An interview with a staff member who was most likely to sell or prescribe medications was conducted. The interview was carried out in Burmese.

During data collection, approximately 80 % of all questionnaires were reviewed by the team supervisor and 15–20 % of all outlets were revisited by a supervisor or/and quality controller for quality control checks.

The core component of the outlet survey is an exhaustive audit of all anti-malarials in stock at the time of the survey. The audit collected product information, including formulation, brand name, active ingredients and strengths, manufacturer, and country of manufacture. The audit additionally collected provider reports on unit cost and amount distributed to individual patients in the previous week. Basic outlet and provider characteristics were collected, including availability of malaria microscopy.

### Ethical approval

The studies were approved by PSI Research Ethical Board (REB) registered under the Office of Human Research Protections (OHRP FWA00009154, IRB#00006978). Local approval was not obtained due to time sensitivities regarding project implementation.

### Data analysis

Data collection was paper-based and entered using CSPro. Double data entry was conducted using Microsoft Access (Microsoft Corp, Redmond, Washington, USA) with built-in range and consistency checks. Data were analyzed across survey rounds using Stata (StataCorp, College Station, TX, USA).

Stata survey settings were used to account for the stratified and clustered sampling strategy. Results were adjusted by sampling weights. Sampling weights were calculated as the inverse of the probability of township selection. Standard indicators were constructed according to definitions applied across the ACTwatch project countries and have been described elsewhere [18, 20]. Briefly, anti-malarials identified during the outlet drug audit were classified according to information on drug formulation, contents and strengths with supporting information including brand or generic name and manufacturer. Among outlets stocking anti-malarials, variables were created to indicate availability by type, including the broad category of ACT, as well as specific categories including: (1) quality-assured ACT (ACT that comply with the Global Fund to Fight AIDS, Tuberculosis and Malaria’s Quality Assurance Policy), non-quality assured ACT, oral artemisinin monotherapy, non-oral artemisinin monotherapy, and non-artemisinin monotherapy.

The volume of anti-malarials recorded in the drug audit were standardized using an adult equivalent treatment dose (AETD) to allow for meaningful comparisons between anti-malarials with different treatment courses. Provider reports on the amount of the drug sold or distributed during the week preceding the survey were used to calculate volumes according to type of anti-malarial. Measures of volume include all dosage forms to provide a complete assessment of anti-malarial market shares. To monitor the extent to which the quality-assured ACT was sold to patients for the recommended 500 Kyat, an indicator was used to capture the percentage of priority outlets that reportedly sold the subsidized ACT at a price less than or equal to 500 Kyat. This price was based on consumer perceptions regarding affordability of anti-malarial treatments and mark-up along the supply chain [14].

## Results

Across the survey rounds the total number of outlets approached for an interview was as follows in the intervention and comparison areas, respectively: 2012, N = 2046 and N = 1612; 2013, N = 1636 and N = 1884; 2014, N = 2939 and N = 2941. Of these, over 95 % of outlets were screened for stocking anti-malarials across the three survey rounds. The total number of anti-malarials audited during each survey was as follows: 2012, N = 3206; 2013, N = 2636; 2014, N = 2396.

### Availability

**Fig. 2:**
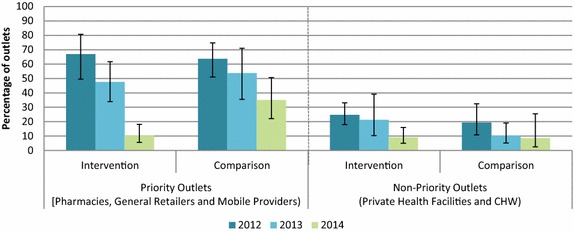
Outlets stocking oral artemisinin monotherapy on the day of survey, 2012, 2013, 2014

**Fig. 3:**
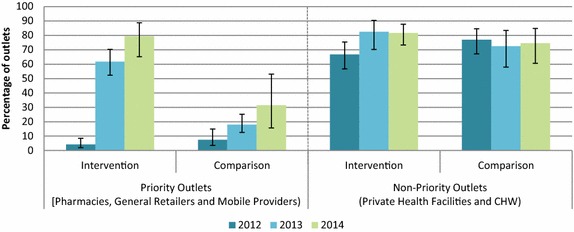
Outlets stocking quality-assured ACT on the day of the survey, 2012, 2013, 2014

Figure 2 shows the availability of oral artemisinin monotherapy among anti-malarial stocking outlet categories in the intervention and comparison areas. The 2012 outlet survey results show that certain outlet types had relatively high availability of oral artemisinin monotherapy (see Fig. 2), and low availability of quality-assured ACT (see Fig. 3), including pharmacies, general retailers and mobile providers. These were categorized as ‘priority outlets’ and targeted for the product promoter intervention. In contrast, private health facilities and community-based health workers had low availability of oral artemisinin monotherapy (<30 %) (see Fig. 2), and higher availability of quality-assured ACT (see Fig. 3). These were categorized as non-priority outlets for the project intervention.

The percentage of priority outlets with oral artemisinin monotherapy in stock on the day of the survey decreased over time in the intervention area from 70 % in 2012 to 10.3 % in 2014. Oral artemisinin monotherapy also declined over time in the comparison area but was less remarkable, with 35.1 % stocking this anti-malarial class in 2014 compared to 63.7 % in 2012. Data trends suggest declining availability of oral artemisinin monotherapy over time among non-priority outlets across both domains at less than 10 % in 2014.

Figure 3 shows the percentage of outlet categories with at least one quality-assured ACT in stock on the day of the survey, among anti-malarial stocking outlets. Quality-assured ACT availability increased among priority outlets (pharmacies, general retailers and mobile providers) between 2012 (4.2 %) and 2013 (61.7 %), and remained high in 2014 (79.4 %). While availability of quality-assured ACT among priority outlets was similar in 2012 across both domains, only one in three priority outlets in the comparison area stocked quality-assured ACT in 2014 (2012 = 7.4 %; 2013 = 18.0 %; 2014 = 31.5 %).

Regarding trend data among non-priority outlets, quality-assured ACT availability among anti-malarial stocking outlet types was generally high and stable over time, and availability ranged between ~70 and ~80 % in 2014.

### Anti-malarial market share

**Fig. 4:**
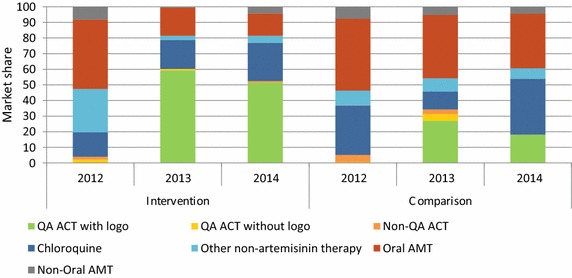
Priority outlet sector market share, by strata

Figure 4 shows the market share of different categories of anti-malarials sold or distributed in the seven days prior to the survey over time according to priority outlets in the intervention and comparison areas. In 2012, oral artemisinin monotherapy accounted for more than 40 % of the anti-malarials distributed by priority outlet types across both domains.

Among priority outlets from the intervention area, oral artemisinin monotherapy (AMT) market share decreased from 44 % in 2012 to 18 % of the total market share in 2013 and 14 % in 2014. Market share of quality-assured (QA) ACT among these priority outlets increased from 3 % in 2012 to 51 % in 2014. Most of the quality-assured ACT that was sold or distributed in 2013 and 2014 across both domains had the lotus leaf logo. Non-artemisinin therapy (mostly chloroquine) accounted for almost one-third of the market share in 2014 among priority outlets in the intervention area, reflecting a decline from 2012 when it held 44 % of the market share.

Among priority outlets in the comparison area, market share of oral artemisinin monotherapy remained high over time, although overall declines were noted (46 %, 2012; 41 %, 2013; 35 %, 2014). Market share of quality-assured ACT was around 30 % in 2013 and declined in 2014 to less than 20 %. In 2014, over 42 % of anti-malarials sold or distributed were non-artemisinin monotherapy.

### Non-Priority outlet sector market share by strata

**Fig. 5:**
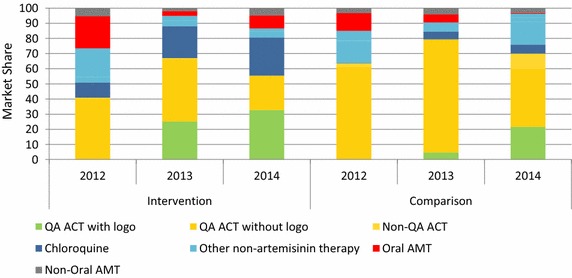
Non-priority outlet sector market share, by strata

In the intervention area among non-priority outlets, oral artemisinin monotherapy accounted for 21 % of the total anti-malarial market share in 2012 and 9 % in 2014 (Fig. 5). Increases in quality-assured ACT market share were observed from 40 % in 2012 to over 50 % in 2014.

In the comparison area among non-priority outlets, oral artemisinin monotherapy accounted for 10 % of the total anti-malarial market share in 2012 and was reportedly no longer sold/distributed in 2014. Quality-assured ACT market share was similar in 2012 and 2014 and ranged between 60–70 % of the total market share.

### Relative market share across priority and non-priority outlet types and categories

**Fig. 6:**
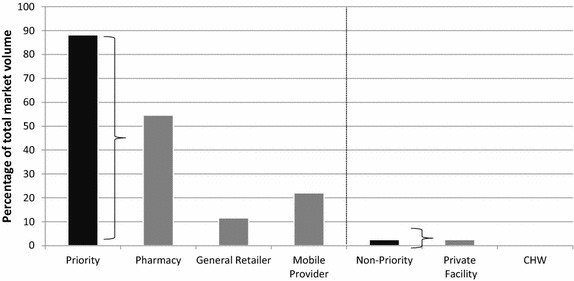
Relative anti-malarial market share in the comparison area, 2014

**Fig. 7:**
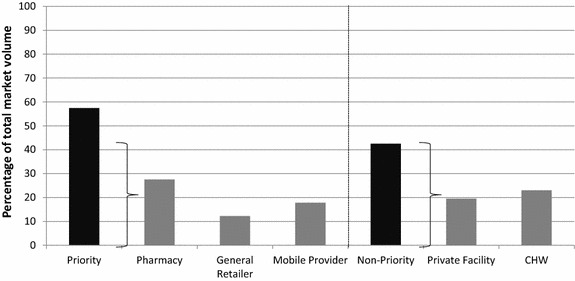
Relative anti-malarial market share in the intervention area, 2014

Figures 6, 7 present the relative anti-malarial market share of all anti-malarials, regardless of anti-malarial class, across each outlet type in 2014. In 2014, the priority outlets accounted for nearly 90 % of all anti-malarials distributed in the comparison areas and nearly 60 % of anti-malarials distributed in the intervention  areas, and anti-malarial distribution was greatest among pharmacies and mobile providers.

### Affordability

**Fig. 8:**
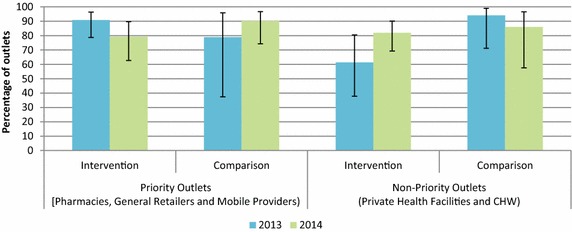
Distribution of *Supa Arte* ® for less than 500 Kyat in 2013 and 2014

The anticipated end users’ price for the PSI-branded, quality-assured ACT was set at 500 Kyat (around $0.75 at the time of the 2012 study). Figure 8 shows the percentage of outlets that distributed the subsidized quality-assured ACT for less than 500 Kyat. The findings show that the majority of priority-sector anti-malarial stocking outlets reportedly sold this subsidized anti-malarial medicine according to the recommended retail price. The results also show that among non-priority outlets, in 2014 most outlets also conformed to the recommended selling price.

## Discussion

The AMTR project to address the removal of oral artemisinin monotherapy from the market has largely been successful as evidenced by substantial reductions in availability and distribution of oral artemisinin monotherapy and increases in availability and distribution of affordable, quality-assured ACT in eastern Myanmar. Data from three outlet surveys confirm substantial improvements between 2012, 2013 and 2014 regarding removal of oral artemisinin monotherapy from the private sector and ensuring high availability, market share and affordability of first-line ACT treatments in eastern Myanmar. These improvements were most notable among priority outlet types, including pharmacies, general retailers and mobile providers. The striking improvements among priority outlet types in intervention areas provide evidence on the importance of supportive interventions in the context of a high-level subsidy. The data also illustrate the importance of priority outlet types as treatment sources for febrile patients in Myanmar, given that most anti-malarials are being sold or distributed through these outlet types in the private sector. Despite successful changes over time, the findings point to the need for further improvements as well as sustained provider and consumer behavior change interventions to ensure optimal access to affordable, quality-assured ACT. The following sub-sections discuss these findings further.

### Anti-malarial availability and market share among priority outlet types

In 2012, the priority outlet types across both the intervention and the comparison areas were poorly performing, with high availability and market share of oral artemisinin monotherapy and negligible availability of quality-assured ACT. The lack of adherence to national malaria guidelines by providers operating in these outlets may be attributed to their lack of formal training and oversight [20]. Information from this baseline assessment provided a key insight regarding the types of outlets that needed additional supportive interventions and that were subsequently targeted as part of the AMTR project.

In 2013 and 2014, two years after the project implementation, substantial increases in the availability of quality-assured ACT and improvements in market share were observed among priority outlet types, particularly in the intervention area. Key improvements observed among anti-malarial stocking pharmacies, general retailers and mobile providers in the intervention area included: (1) dramatic increases in quality-assured ACT availability from less than 10 % in 2012 to nearly 80 % in 2014; (2) increases in market share of quality-assured ACT from less than 5 % in 2012 to over 50 % in 2014, reflecting improvements in ACT availability; (3) declines in the availability of oral artemisinin monotherapy from over 60 % of anti-malarial stocking outlets in 2012 to around 10 % in 2014; and, (4) declines in the market share of oral artemisinin monotherapy from over 40 % in 2012 to 14 % in 2014. While similar trends are observed for priority outlet types in the comparison group, improvements were limited and suggest that simply targeting the supply side through a national subsidy may not be enough to ensure sufficient shifts in anti-malarial markets as a means to achieve adequate coverage and uptake of quality-assured ACT.

The relevance of supportive interventions has been documented elsewhere, namely through the Affordable Medicines Facility for Malaria, implemented in sub-Saharan Africa, which found that private sector subsidies combined with supporting interventions can be effective in rapidly improving availability, price and market share of quality-assured ACT [26], particularly in the private for-profit sector. A core conclusion was that in addition to the availability and distribution of a subsidized ACT, there is an important role for supporting interventions to ensure the success of subsidy programs for public health commodities [27]. The notable market improvements among pharmacies, general retailers and mobile providers in the intervention area suggests the added value of a product promotion intervention, a finding also supported by others [28–30].

Market share of chloroquine hovers around 20 % in the intervention area. This could be explained by the fact that around 26 % of malaria cases in Myanmar are confirmed as vivax malaria [1]. National guidelines stipulate that confirmed vivax malaria cases should be treated with a full course of chloroquine followed by 14 days of primaquine to prevent relapse [1]. It is plausible that the market share of chloroquine is in fact reflective of the readiness of the market to treat vivax cases (although a notable absence of primaquine is recognized). However, in the absence of information on the number of confirmed malaria diagnostic cases, this is difficult to confirm. In addition, while oral artemisinin monotherapy market share has declined, in intervention areas this appears to have been displaced by a rise in non-oral artemisinin monotherapy (which can also increase artemisinin resistance, albeit with lower risk). While non-oral artemisinin monotherapy is for use in severe malaria cases, this situation should continue to be monitored given the inability of the priority outlet providers to adequately test for and treat severe malaria.

While a number of achievements are seen among the priority outlet types, oral artemisinin monotherapy is still commonly sold and accounts for up to 35 % of the market share in the comparison area. While Myanmar has taken regulatory measures to halt the use of oral artemisinin monotherapy, the manufacturing and marketing of these products is still ongoing (and many of these companies are located in China and Vietnam). Persistent sale and distribution of oral artemisinin monotherapy may be explained by the fact that the importation of licensed oral artemisinin monotherapy (products that received a five-year license prior to implementation of the ban) is still permitted as a means to honor existing agreements with companies and manufacturers of this drug. National efforts should be made to enforce the ban on oral artemisinin monotherapy so that this national policy of public health significance is followed.

### The importance of priority outlet types as a source of care

In the context of the findings on availability and market share, it is important to consider the relevance of the priority and non-priority outlet types as a source of anti-malarial care according to the intervention and comparison areas. In 2014, priority outlet types, including pharmacies, general retailers and mobile providers, were responsible for almost 90 % of private sector anti-malarial drug distribution in comparison areas and almost 60 % in the intervention areas. The high relative market share for pharmacies, general retailers and mobile providers indicates the importance of these outlet types as a source of treatment for febrile patients. This finding is further supported by household surveys which have found that most people in Myanmar seek treatment in the private sector, and most likely from general retailers, mobile providers and pharmacies [19, 31], indicating the relevance of these outlet types as a source of treatment, in addition to community health workers and health facilities.

### Anti-malarial availability and market share among non-priority outlet types

The levels of ACT availability among non-priority outlets remained relatively high and stable over time, and market share data illustrate that ACT was the most commonly distributed anti-malarial, although slight declines were observed between 2013 and 2014 in both the intervention and comparison area. Incremental increases were also observed regarding the market share of ACT with the logo, and in 2014 ACT with the logo comprised about 30 % of market share in the intervention area and 20 % in the comparison area. While these improvements are only moderate, it is suggestive of market penetration and uptake of subsidized ACT, particularly in the intervention area. While availability of oral artemisinin monotherapy was relatively low at baseline, general declines were also observed among these outlets so that by 2014, less than 10 % had oral artemisinin monotherapy in stock on the day of survey.

The performance of the non-priority outlets is quite consistent with evidence from other countries which illustrates that regulated outlets (such as private facilities) generally perform better than other private sector outlet types [19, 21]. Indeed, in Myanmar several supportive measures have been made to reach these outlet types and providers through government and other NGO efforts, where private health facility providers are trained in accordance with national policies and operate in specific sites and locations designed by the Government of Myanmar [11]. The findings from 2014 indicate widespread market share of quality-assured ACT as well as distribution and sales of treatment for vivax malaria, particularly in the comparison areas, suggesting the readiness of the priority outlets to provide malaria treatment according to national guidelines.

### Price

A key premise of the AMTR project was that promoting maximum coverage of quality-assured ACT in the private sector would lead to market competition among outlets and help to control the price paid by consumers. The anticipated retail price of the quality-assured ACT was 500 Kyat. The findings show that most of the priority and non-priority outlets stocking the quality-assured ACT have adhered to this guideline. Higher availability and market share of quality-assured ACT over time and reductions in oral artemisinin monotherapy thus supports the initial premise of the AMTR project in ensuring subsidized, quality-assured ACT in the supply chain would effectively squeeze out oral artemisinin monotherapy due to price competition. This is attributed to a multi-pronged approach, including the efficiency of private sector supply chain, high coverage and availability of quality-assured ACT, and constant supply, as well as high-level donor subsidy. Similar findings have been found in other countries with relatively high rates of oral artemisinin market share, namely Cambodia and Nigeria, where the introduction of subsidized ACT in the market complemented by other supporting interventions, led to increases in quality-assured ACT and declines in oral artemisinin monotherapy [26, 31].

### Future activities and research

Given declining malaria prevalence rates, as well as the confirmation of the spread of artemisinin resistance along the western border of Myanmar, the deployment of rapid diagnostic tests (RDT) in the private sector would now be beneficial to avoid drug wastage, to improve case management and to prevent development of resistance to partner drugs. An evaluation of the quality of the drugs, specifically the quality-assured ACT, that are available in the market should also be conducted as a means to ensure that no falsified medicines are in circulation, given that sub-standard or fake ACT are a threat to malaria elimination and will lead to drug resistance.

### Limitations

This study had several limitations, some which are inherent to the ACTwatch outlet surveys and have been documented elsewhere [20, 21]. Specific to the design of this research serving as an evaluation of the AMTR project, there was a lack of randomization for intervention and comparison areas. The lack of a randomized control group constrains the ability to assess precisely the degree to which the changes in availability, price and market share are attributable to the AMTR project or to the specific provider behavior change strategies that were implemented in the intervention areas through the product promoters. Documentation of other interventions identified a range of factors that could also have improved outcomes, including policy changes banning importation and distribution of oral artemisinin monotherapy in 2012. Other activities that could have contributed to change include other supporting interventions implemented by partners. As such, caution is merited in making causal inferences about the impact of the AMTR project and the changes. Finally, the outlet survey reports on relative volumes distributed in terms of full course treatments (i.e., AETD). While declining volumes of oral artemisinin monotherapy relative to quality-assured ACT are observed, it is acknowledged that people may receive doses of this drug class that are not full course adult treatments. It is therefore plausible that many more people will be receiving small doses of oral artemisinin monotherapy, which is not well reflected in market share.

### Key lessons

There are a number of important lessons that can be taken from the AMTR project. In terms of speed, the project demonstrated significant improvements in the anti-malarial market landscape in a relatively short period of time. This was essential in order to deal with the rapidly changing epidemiology and fears of further spread of artemisinin resistance in 2012. In addition, the project addressed this at scale to ensure that quality-assured ACT was made available throughout the country and not just confined to the eastern border. Utilizing the existing private sector supply system rather than creating a parallel system was an integral component of this, and, without it, it is doubtful whether the project would have been successful in a country such as Myanmar, where there are more than 130 ethnic groups, ethnic conflicts and civil unrest.

## Conclusion

In 2012, the widespread availability and use of oral artemisinin monotherapy in Myanmar posed a significant threat to malaria elimination efforts in the region. Evidence from outlet surveys demonstrated the successful design and implementation of a strategy to rapidly remove oral artemisinin monotherapy from pharmacies, general retailers and mobile providers and to replace its use with quality-assured ACT. It demonstrated the critical role of the private sector as a source of treatment in Myanmar. To this end, the NMCP must continue to engage with and monitor the private sector as a means to ensure elimination goals are met and treatments are administered according to national policy. In line with recent epidemiological evidence, the next steps for the AMTR project are to focus on rational drug use and appropriate treatment for falciparum and vivax malaria by increasing access to affordable RDT.

## Additional file

10.1186/s12936-016-1292-8 Outlet survey Myanmar questionnaire, 2014. This file includes the full questionnaire administered during Myanmar’s 2014 outlet survey.
