# Supplementary material for: Oral artemisinin monotherapy removal from the private sector in Eastern Myanmar between 2012 and 2014
Source: Malar J. 2016 May 23;15:286. doi: 10.1186/s12936-016-1292-8 (PMC4877749; doi:10.1186/s12936-016-1292-8)
Supplement: Supplementary file 1 — 10.1186/s12936-016-1292-8 Outlet survey Myanmar questionnaire, 2014. This file includes the full questionnaire administered during Myanmar’s 2014 outlet survey. [file 12936_2016_1292_MOESM1_ESM.docx]

# ACTwatch outlet survey questionnaire

***Section I: Census Information (Interviewer to complete this section for all outlets)***

| **Outlet ID**  Interviewer-Township-Ward/Village tract-Outlet Code: | | | | [___\|___]-[___\|___]-[___\|___\|___]-[___\|___\|___] | | |
| --- | --- | --- | --- | --- | --- | --- |
| C1. Today’s date (DD/MM/YYYY) | | | | [___\|___]-[___\|___]-[_2_\|_0_\|_1_\|_4_] | | |
| C2. Interviewer’s name [_____________________________] | | | | C2a. Interviewer’s code [___\|___] | | |
| C3. Division/State [______________________] | | | | C3a. Division/State code [___\|___] | | |
| C4. Township [___________________________] | | | | C4a. Township code [___\|___] | | |
| C5. Ward/Village tract [_____________________________] | | | | C5a. Ward/Village tract Code [___\|___\|___] | | |
| C6. Village [_____________________________] | | | | C6a. Village code [___\|___\|___] | | |
| C7. Name of outlet [_____________________________]  ***If no name, record “no name” or owner’s name*** | | | | C7a. Outlet code [___\|___\|___] | | |
| ***C8. Type of Outlet*** | | | | | | |
| 1 Private Hospital | | 5 SPH | | 9 General store/Convenient store | | |
| 2 Poly Clinic | | 6 Government Health Staff (Specify):  [________________________] | | 10 Village shop | | |
| 3 Non-SQHC Clinic (GP) | | 7 Informal Provider (Quack) | | 96 Other (specify)  [____________________________________] | | |
| 4 SQHC clinic | | 8 Pharmacy/drug shop (specify):  ***(circle only one)***  8a. mainly whole sale  8b. mainly retail | |  | | |
| ***Interviewer enters outlets.*** Hello, My name is [*interviewers name*], and I work for PSI/Myanmar. We are conducting a study on the availability of antimalarial medicines. The results will be used to improve the availability of appropriate antimalarial treatment in Myanmar. I would like to ask you a few questions to see if you could be part of the study.  ***(Interviewer to read the verbal consent form aloud to the participant here.)*** | | | | | | |
| ***Screening*** | | | | | | |
| Sr | Questionnaire | | Response | | Code | Skip |
| S1 | Do you have any antimalarial medicines in stock today?  ***If necessary, prompt with common antimalarial names. If necessary, prompt those antimalarials are for provision/sale to patients***. | | Yes  No | | 1  0 | *Provide information on study and gain consent. Record start time in C10, then go to Tablet Audit Sheet.*  *0🡪 go to S2* |
| S2 | Are there any antimalarial medicines that are out of stock today, but that you stocked in the past **3 months?** | | Yes  No    Don’t know | | 1  0  99 | *Provide information on study and gain consent. Record start time in C10 and go to Q13.*  *0,99🡪 go to S3* |
| S3 | Are you offering any diagnostic services or selling any diagnostic tests for suspected malaria | | Yes | | 1 | *Provide information on study and gain consent. Record start time in C10 and go to Q15.* |
|  |  | | No | | 0 | *0,🡪 Go to C10 and complete Result of Visit, then record details in Ending the Interview.* |

| ***Result of Visits*** | | |
| --- | --- | --- |
| C10. ***Interviewer record result of visit(s)*** | | |
|  | **Visit 1** | **Visit 2** |
| Date (dd/mm/yy) | [__\|__]-[__\|__]-[__\|__] | [__\|__]-[__\|__]-[__\|__] |
| Time started  ***(use 24hr clock)***  ***95:95 = NA*** | [___\|___]:[___\|___] | [___\|___]:[___\|___] |
| Time completed  ***(use 24hr clock)***  ***95:95 = NA*** | [___\|___]:[___\|___] | [___\|___]:[___\|___] |
| Result | [___] | [___]  [___] |
|  | 1 = Completed (Provider interview conducted)🡪*Go to E1*  2 = Outlet does not meet screening criteria🡪*Go to E1*  3 = Interview interrupted 🡪*Go to C12 and note time convenient for call back*  4 = Eligible respondent not available🡪*Go to C12 and note time convenient for call back*  5 = Outlet not open at the time🡪*Go to C12 and note time convenient for call back*  6 = Outlet closed permanently🡪*Go to E1*  7 = Refused🡪*Go to C11*  8 = Other ***(specify):*** [_________________] | |

| ***Refusal / Appointments*** |
| --- |
| C11. If the provider refused, why? ***Circle one answer.***  1 = Client load ***Ask for a time provider would prefer to be interviewed, note in C12 and return at this time.***  2 = Thinks it’s an inspection / nervous about license ***Go to E1***  3 = Not interested ***Go to E1***  4 = Refuses to give reason ***Go to E1***  5 = Other ***(specify): Go to E1*** |
| C12. ***Interviewer: use this space to record any appointment that has been made for a call back to complete the interview*** |

| ***Section VII: Ending the Interview*** |
| --- |
| E1. Physical address or location identifiers of outlet (not PO box)  ***(Give detailed description that will help to find the outlet)*** |
| E2. Telephone number  [___\|___\|___\|___\|___\|___\|___\|___\|___\|___]  ***9999999995 = Not applicable/no respondent/no telephone***  ***9999999997 = Refused*** |
| E3. Do you have any questions or comments for us? ***Record any questions or comments from provider.*** |
| E4. ***Additional observations by interviewer (if any)*** |

***THANK THE PROVIDER AND END INTERVIEW***

| ***Section II: Antimalarial Audit (Interviewer to follow instructions outlined on this page)*** |
| --- |
| A1*.* Can you please show me the full range of antimalarials that you currently have in stock.  Do you currently have any of the following antimalarials in stock?  ***Prompt entire list using antimalarial prompt card. No response to be recorded.***   - Artemether lumefantrine, such as *Coartem20/120,* ***Artemether and lumefantine , Coartem Dispersible , Artefam 20/120, lumartem*** - Artesunate amodiaquine, such as *Artemodi (Adults/Children), Quinsunat, Arsuamoon, Co-Artesun , Macsunate FD(kid)* - Other artemisinin combination therapies, such as *Duo-cotecxin, D-Artepp, Arco, Artecospe(Adults), Artecom, Arfloquine* - Artemisinin monotherapies, such as *AA Artesunate (tab), AA Artemether, Artesunate injection, Artemedine, Aretemether, Lurither, Traphasunate, Artesun, Arcomether, Glinther, Betamotil, Falcinate, Artim 80, Arthesis, EMAL, Artesiane 80, Artemether injection* - Artemether, such as *Artem, AA-Armether , Armether , Artemedine , Betamotil* - Artesunate, such as *AA -Artesunate , Artesunate(tablets) , Traphasunate , Falcinate , Arthesis* - Chloroquine, such as *Chloroquine tablets , Chlorofos, Chloroquine Phosphate , Paraquine , Tabellaechloro-quin, Jasochlor, Malacin, Chloroquine* - SP, such as *Pyrixine, Malidar,SP* - Quinine, such as *quinine tablets,quinine sulphate , Jasoquin* - Mefloquine, such as *Mefloquine* - Injectables, such as *Artem, Quinine Dihydrochloride, Quinine (Injection), Artesunate for Injection, Artemedine, Larither,Artesun, Arcomether,Pekquine Injection, Glinther, Betamotil, Artim 80, Malacin, EMAL, Artesiane 80, Artemether injection* - Granules or powders, such as *Artesunate for Injection, Artim 80*   ***If the outlet has no antimalarials in stock, go to Question 13*** |
| ***Interviewer to separate the antimalarials into two piles:***   - ***The first pile should contain all the antimalarials in the form of tablets, suppositories, or granules. Use the Tablets, Suppositories & Granules Drug Audit Sheet to record these.*** - ***The second pile should contain all the antimalarials in any form other than tablets, suppositories or granules. Use the Non-Tablet Drug Audit Sheet to record these.*** |
| ***Interviewer to proceed to the drug audit.***  ***Different Drug Audit sheets should be used to record the product information based on the dosage form of the medicine.***  ***If additional audit sheets are needed add these sheets after the ones provided and staple the questionnaire again.***  ***Number each drug by assigning a product number***  ***Number each audit sheet in the bottom of the page***  ***All pages should be in order before you move onto the next outlet.*** |

**Tablet, Suppository and Granule Audit Sheet [___|___]-[___|___]-[___|___|___]-[___|___|___]**

| **Product number**  [__\|__] | [__\|__]  [__\|__]  [__\|__] | **1. Generic name** | | | **2. Strength**  [__\|__\|__].[__]mg  [__\|__\|__].[__]mg  [__\|__\|__].[__]mg | | **2a. Is this base strength?**  [__]  1 = Yes  [__] 0 = No  8 = Don’t know  [__]  ***If no, specif salt:***  [_________________] | **3. Dosage form**  1 = Tablet  2=Suppository  3 = Granule  [___] | **4. Brand name** | | **5. Manufacturer** | **6. Country of manufacture**  Do not write here |
| --- | --- | --- | --- | --- | --- | --- | --- | --- | --- | --- | --- | --- |
|  | [__\|__] | | | |  |  |  |  |  |  |  | [__\|__\|__] |
| **7. Package size**  There are a total of  [___\|___\|___\|___] tablets / suppositories / granule packs in each:  1 = Package  2 = Pot/tin  [___] | | | **8. Is product a fixed-dose combination (FDC)**  1 = Yes  0 = No  8 = Don’t   know  [___] | **9. Does product have the Padonma logo?**  1 = Yes  0 = No  [___] | | **10. Amount sold/distributed in the last 7 days to individual consumers** (*Record # of packages / tins described in* Q7 *OR record the total # of tablets / suppositories / granule packs sold*)  This outlet sold [___\|___\|___] packages/tins in the last 7 days  OR  This outlet sold [___\|___\|___] tablets/ suppositories or granule packs in the last 7 days  ***Not applicable = 995; Refused = 997;***  ***Don’t know = 998*** | | **10a. Has product been stocked out at any time in past 2 weeks?**  1 = Yes  0 = No  8 = Don’t   know  [___] | **10b. Has product been stocked out at any time in past 3 months?**  1 = Yes  0 = No  8 = Don’t   know  [___] | **11. Retail selling price**  [___\|___\|___\|___] tablets, suppositories or granule packs cost an individual customer  [___\|___\|___\|___\|___] KYAT | **12. Wholesale purchase price**  For the outlet’s most recent wholesale purchase  [___\|___\|___\|___]  tablets, suppositories or granule packs cost  [___\|___\|___\|___\|___]  KYAT | **13. Comments** |
|  |  |  |  |  |  |  |  |  | **10c. The stock out period in past 3 month**  **(Ask only those who answered “1” in 10b)**  1. <1week  2. ≥1week  8. Don’t know  [___] |  |  |  |
|  |  |  |  |  |  |  |  |  |  | ***Free = 00000; Refused = 99997;Don’t know = 99998*** | |  |

**Tablet, Suppository and Granule Audit Sheet [__|__] of [__|__]**

**Non-Tablet Drug Audit Sheet (NT): syrup, suspension, injections & others**

| **Product number**  [__\|__] | [__\|__]  [__\|__]  [__\|__] | | **1. Generic name** | **2. Strength**  [__\|__\|__\|__].[__]mg/[__\|__\|__].[__]mL  [__\|__\|__\|__].[__]mg/[__\|__\|__].[__]mL  [__\|__\|__\|__].[__]mg/[__\|__\|__].[__]mL  (*Note: no mL recorded for powder injection*) | | | **2a. Is this base strength?**  [__] 1 = Yes  [__] 0 = No  [__] 8 = Don’t know  ***If no, specify salt:***  [_______________] | **3. Dosage form**  1 = Syrup  2 = Suspension  3 = Liquid inj.  4 = Powder inj.  6 = Other ***(specify)***  [___________] | **4. Brand name** | | **5. Manufacturer** | |
| --- | --- | --- | --- | --- | --- | --- | --- | --- | --- | --- | --- | --- |
|  | [__\|__] | | |  |  |  |  |  |  |  |  |  |
| **6. Country of manufacture**  Do not write here | | **7. Package size**  There are a total of  [___\|___\|___\|___].[__] mL  (or mg for powder injections) in each:  1 = Bottle  2 = Ampoule/vial  [___] | | **8. Does this product have the Padonma logo?**  1 = Yes  0 = No  [___] | **9. Amount sold/ distributed in the last 7 days to individual consumers**  This outlet sold  [___\|___\|___\|___] bottles, ampoules or vials in the  last 7 days  ***Refused = 9997;***  ***Don’t know = 9998*** | **10a. Has product been stocked out at any time in the past 2 weeks?**  1 = Yes  0 = No  8 = Don’t   know  [___] | | **10b. Has product been stocked out at any time in the past 3 months?**  1 = Yes  0 = No  8 = Don’t know  [___] | **11. Retail selling price**  [___\|___\|___] bottles ampoules or vials cost an individual customer  [___\|___\|___\|___\|___]  KYAT | **12. Wholesale purchase price**  For the outlet’s most recent wholesale purchase:  [___\|___\|___\|___] bottles, ampoules or vials cost  [___\|___\|___\|___\|___]  KYAT | | **13. Comments** |
|  |  |  |  |  |  |  |  | **10c. The stock out period in past 3 month**  **(Ask only those who answered “1” in 10b)**  1. <1week  2. ≥1week  8. Don’t know  [___] |  |  |  |  |
| [__\|__\|__] | |  |  |  |  |  |  |  | ***Free= 00000; Refused = 99997;Don’t know=99998*** | | |  |

**Non-Tablet Drug Audit Sheet (NT): syrup, suspension, injections & others** [__|__] of [__|__]

| Sr | Questionnaire | Response | Code | Skip |
| --- | --- | --- | --- | --- |
| 13 | Are there any antimalarial medicines that are out of stock today, but that you stocked in the past **2 weeks**? | Yes  No  Don’t know | 1  0  99 | 0,99🡪Q14 |
| 13a | Do you know the names of these treatments? (Use Show Card to help the provider to memorize) | Yes  [_____________]  [_____________]  [_____________]  No | 1  0 | *Specify below, record one medicine per line. Will accept generic or brand names.* |
| 14 | Are there any antimalarial medicines that are out of stock today, but that you stocked in the past **3 months**? | Yes  No  Don’t know | 1  0  99 | 0,99🡪Q15 |
| 14a | Do you know the names of these treatments? (Use Show Card to help the provider to memorize) | Yes  [_____________]  [_____________]  [_____________]  No | 1  0 | *Specify below, record one medicine per line. Will accept generic or brand names.* |

| ***Microscopy*** | | | | |
| --- | --- | --- | --- | --- |
| 15 | Is malaria microscopic testing available here today? | Yes  No | 1  0 | 0🡪Q16 |
| 15a | Please show me the microscopic test that is available in this outlet. ***(Ask for the permission to see the microscopic test.)*** ***Interviewer: Is the microscopic test observed?*** | Yes  No | 1  0 |  |
| 15b | How much do you charge for a microscopic test for malaria? | [___\|___\|___\|___\|___]Kyats  ***00000 = Free; 99999 = Don’t know*** |  |  |
| 15c | How many microscopic tests for malaria were conducted in this outlet over the past 7 days? | [___\|___\|___]  ***999 = Don’t know*** |  |  |
| 15d | Including the owner and yourself, have any staff members in this outlet been trained to prepare a blood slide and read the results of a microscopic test for malaria? | Yes  No | 1  0 |  |
| ***Section III: RDT Audit*** | | | | |
| Sr | Questionnaire | Response | Code | Skip |
| 16 | Are malaria rapid diagnostic test kits (RDTs) available here today? | Yes  No | 1  0 | 0🡪Q17 |
| 16a | Please show me the full range of RDTs that you currently have in stock. **Do you currently have any of the following?**  ***Read entire list. No response to be recorded.***  ***Proceed to the RDT audit. If additional audit sheets are used, add these sheets after the ones provided and staple the questionnaire again. All pages should be in order before you move onto the next outlet.*** | SD Bioline P.f/P.v  SD Bioline P.f/Pan  First Response  Care Start  Accurate  Clungene  ParaHit  Others (specify) [___________________] | 1  2  3  4  5  6  7  96 |  |

**Rapid Diagnostic Test Audit Sheet (RDT)** **[___|___]-[___|___]-[___|___|___]-[___|___|___]**

| **Product**  **number**  [__\|__] | **1. Brand name** | | **1a. Antigen test** *(circle ALL that apply)* | | | **1b. Parasite species**  *(circle ALL that apply)* | | **2. Manufacturer** | **3. Country of Manufacture**  ***Not indicated = 998*** | **4. Lot Number** | **5. Number of tests sold/ distributed /used in the last 7 days to individual consumers**  *(Record total # of tests)*  This outlet sold or distributed  [___\|___\|___\|___] tests in the last 7 days  ***Refused = 9997 ; Don’t know=9998*** | |
| --- | --- | --- | --- | --- | --- | --- | --- | --- | --- | --- | --- | --- |
|  |  |  | Not indicated  HRP2  pLDH  Aldolase | | **Z**  **A**  **B**  **C** | Not indicated Pf  Pv  Po  pan  vom/Pvom | **Z**  **A**  **B**  **C**  **D**  **E** |  |  |  |  |  |
|  |  |  |  |  |  |  |  |  | [___\|___\|___] |  |  |  |
| **6a. Has this test been stocked out at any time in the past 2 weeks?**  1 = Yes  0 = No  8 = Don’t know  [___] | | **6b. Has this test been stocked out at any time in the past 3 months?**  1=Yes  0 = No  8 = Don’t know  [___] | | **7. Price for adults**  For an adult who needs a test, how much do you charge:  To buy the test: [___\|___\|___\|___\|___] KYAT  For consultation fees: [___\|___\|___\|___\|___] KYAT  For other fees ***(specify)***: [___\|___\|___\|___\|___] KYAT  [______________________________________] | | | | | **8. Price for children under 5**  For a child under five who needs a test, how much do you charge:  To buy the test:  [___\|___\|___\|___\|___] KYAT  For consultation fees: [___\|___\|___\|___\|___] KYAT  For other fees ***(specify)***:  [___\|___\|___\|___\|___] KYAT  [_____________________________________] | | **9. Wholesale purchase price**  For the outlet’s most recent wholesale purchase:  [___\|___\|___\|___] tests cost  [___\|___\|___\|___\|___] KYAT | **10. Comments** |
| ***Free = 00000; NA = 99995; Refused = 99997; Don’t know=99998*** | | | | | | | | | | | |  |

RDT Audit Sheet [___|___] of [___|___]

| Sr | Questionnaire | Response | Code | Skip |
| --- | --- | --- | --- | --- |
| 17 | Are there any RDTs that are out of stock today, but that you stocked in the past **2 weeks**? | Yes  No  Don’t know | 1  0  99 | 0,99🡪18 |
| 17a | Do you know the names of these RDTs? | Yes  [_____________]  [_____________]  [_____________]  No | 1  0 | Specify below, record one RDT per line. |
| 18 | Are there any RDTs that are out of stock today, but that you stocked in the past **3 months**? | Yes  No  Don’t know | 1  0  99 | 0,99🡪Q P1 |
| 18a | Do you know the names of these RDTs? | Yes  [_____________]  [_____________]  [_____________]  No | 1  0 | Specify below, record one RDT per line. |

***Section IV: Provider Questionnaire***

| Sr | Questionnaire | | Response | Code | Skip |
| --- | --- | --- | --- | --- | --- |
| P1 | What is your job at this outlet?  ***Do not read list. Multiple responses allowed.*** | | Medical doctor  Owner  Nurse  Clinic assistant  Shop assistant  Relative of the owner  Other *(specify)* [_______________] | **MR**  1  2  3  4  5  6  96 |  |
| P1a | For how many years have you worked in this outlet?  ***If less than 1 year, enter “01”*** | | [___\|___] years |  |  |
| P1b | What is the highest level of education you completed? | | No schooling  Monastic or primary grade  Middle Grade  High Grade  Passed matriculation  Diploma or degree  Post-grad | 1  2  3  4  5  6  7 |  |
| P1c | Do you have any of the following health qualifications? | | No health qualifications  Pharmacist  Laboratory technician  Health assistant  Medical doctor  Nurse / Midwife  PHS Compounder  Pharmacist trained by private agency  Other (specify) [_________________] | 0  1  2  3  4  5  6  7  8  96 |  |
| P2a | In the past 12 months, have you attended any trainings or workshops about malaria diagnosis (RDT or microscopy)? | | Yes  No  Don’t know | 1  0  99 |  |
| P2b | In the past 12 months, have you attended any trainings or workshops about malaria treatment, such as how to dispense medicines; proper dosing of medicines; case management? | | Yes  No  Don’t know | 1  0  99 |  |
| P3 | Including the owner and yourself, how many people work here?  ***If outlet has multiple dispensaries, record number of workers at this dispensary only***. | | [___\|___]  Don’t know | 99 |  |
| P4 | Of all the people who work here, how many prescribe or dispense medicines? | | [___\|___]  Don’t know | 99 |  |
| P5 | What is the highest level of education among the people working in this outlet?  ***(Prompted. Circle one response)*** | | No schooling  Monastic or primary grade  Middle Grade  High Grade  Passed matriculation  Diploma or degree  Post-grad | 1  2  3  4  5  6  7 |  |
| P6 | **Not** including yourself, does anyone working in this outlet have a health-related qualification? | | Yes  No  Don’t know | 1  0  99 | 0,99🡪 P8 |
| P7 | **Not** including yourself, how many people working in this outlet (including the owner) have the following types of health qualifications?  ***Read list. Enter ‘00’ if the answer is ‘none.’*** | | Pharmacist  Laboratory technician  Health assistant  Medical doctor  Nurse / Midwife  PHS Compounder  Pharmacist trained by private agency  Other (specify) [_________________] | [___\|___]  [___\|___]  [___\|___]  [___\|___]  [___\|___]  [___\|___]  [___\|___]  [___\|___]  [___\|___] |  |
| P8 | Do you have a license/temporary license to sell drugs? | | Yes  No | 1  0 | 0🡪P10 |
| P9 | ***Interviewer observes the license and record response based on observation.*** | | Yes, license physically observed  No, license not physically observed | 1  0 |  |
| P10 | **(Do not ask this question if the outlet is clinic)**  Do you know *P.falciparum* and *P.vivax* malaria? | | Yes  No | 1  0 | 1 🡪 P10a  0 🡪 P11 |
| P10a | Do you treat *P.falciparum* differently compared to *P.vivax*? | | Yes  No | 1  0 | 1  0 |
| P11 | In your opinion, for treating uncomplicated malaria, what is the most effective antimalarial medicine?  ***Looking for either Generic name or Brand name. Ask provider to show you the medicine if in stock.*** | | [_____________________________]  Cocktail  Don’t know | 1  99 |  |
| P11a | What antimalarial medicine for treating uncomplicated malaria, do you most often recommend to customers?  ***Looking for either Generic name or Brand name. Ask provider to show you the medicine if in stock.*** | | [_______________________________]  Cocktail  Don’t know | 1  99 |  |
| P12 | **(Do not ask this question if the outlet is clinic)**  In your opinion, for treating uncomplicated **P. falciparum**, what is the most effective antimalarial medicine?  **Looking for either Generic name or Brand name. Ask provider to show you the medicine if in stock.** | | [________________________]  Cocktail  Don’t know | 1  99 |  |
| P12a | **(Do not ask this question if the outlet is clinic)**  In your opinion, for treating uncomplicated **P. vivax**, what is the most effective antimalarial medicine?  **Looking for either Generic name or Brand name. Ask provider to show you the medicine if in stock.** | | [_______________________]  Cocktail  Don’t know | 1  99 |  |
| P13 | What antimalarial medicine for treating uncomplicated **P. falciparum**, do you most often recommend to customers?  **Looking for either Generic name or Brand name. Ask provider to show you the medicine if in stock.** | | [________________________]  Cocktail  N/A; Don’t recommend | 1  99 |  |
| P13a | What antimalarial medicine for treating uncomplicated **P. vivax**, do you most often recommend to customers?  **Looking for either Generic name or Brand name. Ask provider to show you the medicine if in stock.** | | [_______________________]  Cocktail  N/A; Don’t recommend | 1  99 |  |
| P14 | | How do you typically decide which antimalarials to stock?  Read list. Multiple responses allowed. | Most profitable  Recommended by government  Lowest priced  Drug company/sales rep influence  Consumer demand  Brand reputation  Dosage form  Easily available  Prescribed most often by doctors  Provided by PSI  More effective  Other (specify) [________________]  Don’t know | MR  1  2  3  4  5  6  7  8  9  10  11  96  99 |  |

| P14a | | Which antimalarials provide a good profit margin for you?  ***Looking for either Generic name or Brand name.*** | [_________________________________]  [_________________________________]  [_________________________________]  All antimalarials are the same  Refuse to answer  Don’t know | 1  2  99 |  |
| --- | --- | --- | --- | --- | --- |
| P15 | | Do your customers know ask for anti malarial medicines by name?  ***Read list. One response only.*** | Yes  No  No, they have a written prescription  Don’t know | 1  0  2  99 | 1 🡪 P15a  0 🡪 P16  2 🡪 P15b  99 🡪 P16 |
| P15a | | What are the three most common antimalarial drugs that people ask for by name?  ***Looking for either Generic name or Brand name or Cocktail. Ask provider to show you the medicine if in stock. 99 if don’t know.*** | [__________________________________]  [__________________________________]  [__________________________________] |  | Skip to P16 |
| P15b | | What are the three most common antimalarial drugs that were prescribed by providers (came to you through prescription notes)?  ***Looking for either Generic name or Brand name or Cocktail. Ask provider to show you the medicine if in stock. 99 if don’t know.*** | [__________________________________]  [__________________________________]  [__________________________________] |  |  |
| P16 | | Do you normally decide which antimalarial medicines customers receive?  ***Read list. One response only.*** | Yes  No  No, they have a written prescription  Don’t know | 1  *0*  *2*  *99* |  |
| P17 | | In the past **month**, have customers bought antimalarials on credit? | Yes  No  Don’t know | 1  0  99 | 0,99 🡪 P18 |
| P17a | In the past  **month**, how many customers have bought antimalarials on credit? | | [___\|___\|___]    ***999 = Don’t know*** |  |  |
| P18 | In the past **month**, did you ever cut blister packs or sell partial courses of antimalarials? | | Yes  No  Don’t know | 1  0  99 | 0,99🡪P19 |
| P18a | What is/are the reason(s) that you cut blisters or sell partial courses?  ***Do not read list. Probe for anything else. Multiple responses.*** | | Customers/Patients’ request  Cut/partial is sufficient  I have small / insufficient stock  Customers/Patients cannot afford full blister/pack  Makes it easier for the patient to take medicine  Other(specify))[_______________]  Don’t know | **MR**  1  2  3  4  5  96  99 |  |

| P19 | Please name the first-line medicine recommended by the government (National Malaria Program/VBDC) to treat uncomplicated *p. falciparum* malaria? | [___ ______________]  Don’t know | 99 |  |
| --- | --- | --- | --- | --- |
| P20 | When do you refer your customers/ patients with suspected malaria to the nearest health facility?  ***Don’t read answers. Multiple responses allowed.*** | Don’t refer  Pregnant mother  Children under age of 1 year  Fever not subside  when I think severe malaria  Loss of consciousness/ Coma  In Fits (Convulsion)  Unable to sit/ eat/ drink  Frequent vomiting  Restlessness  Jaundice or very pale  Black color urine or little or no urine  Other ***(specify)*** [______________]  Don’t know | **MR**  0  1  2  3  4  5  6  7  8  9  10  11  96  99 |  |
| P21 | Who is at risk of getting malaria in Myanmar?  ***Don’t read answers. Multiple responses allowed.*** | Forest related worker  Migrant people/worker  Plantation worker  Gold/jade/gem miner  Pregnant woman  Children under 5  Other ***(specify)*** [______________]  Don’t know | **MR**  1  2  3  4  5  6  96  99 |  |
| P22 | Is malaria testing service using RDT available here?  ***Show RDT images in prompt card.*** | Yes  No | 1  0 | 0 🡪P27 |

| Sr | | Questionnaire | | Response | | | Code | | | Skip | |
| --- | --- | --- | --- | --- | --- | --- | --- | --- | --- | --- | --- |
| P23a | | Did anyone from this outlet (including you) receive training on how to use RDT? | | Yes  No | | | 1  0 | | |  | |
| P23b | | If yes to P23a, who received that training? | | Myself (respondent)  Medical doctor  Owner  Nurse  Clinic assistant  Shop assistant  Relative of the owner  Other *(specify)* [_______________] | | | 1  2  3  4  5  6  7  96 | | |  | |
| P24a | | How often do you test people who have fever for malaria using a blood test? | | Always  Most of the time  Sometimes  Rarely  Never | | | 1  2  3  4  5 | | | 5 🡪P25 | |
| P24b | | Did the last patient you provided an antimalarial to also receive a malaria diagnostic test from this outlet? | | Yes  No  Don’t know | | | 1  0  99 | | | If the respondent answered “1” in P24a 🡪Skip to P26 | |
| P25 | | What is the **main** reason that you would not test a client with fever for malaria using a blood test?  **(circle one)** | | Do not have tests in stock  Do not think is necessary  Customers do not want a test  Customers cannot afford a test  I don’t know how to do that  Other (specify) ____________________ | | 1  2  3  4  5  96 | | |  | | |
| P26 | | When an RDT is positive for malaria, how likely do you think it is that the person tested actually has malaria?  ***Read list. Record only one response.*** | | Certain they have malaria  Very likely they have malaria  Somewhat likely they have malaria  Not very likely they have malaria  Not at all likely they have malaria  Don’t know | | 1  2  3  4  5  99 | | |  | | |
| P27 | | In your opinion, how important is it for a person with fever to get tested to confirm malaria before treatment? | | Very important  Somewhat important  Not very important  Not at all important  Don’t know | | 1  2  3  4  99 | | | 1, 2, 99 🡪 P29  3,4 🡪 P28 | | |
| P28 | | If answered 3 or 4 in P27, why do you think it is not important to provide a test for a person with fever before giving malaria treatment? | | [_________________________________] | |  | | |  | | |
|  | |  | |  | |  | | |  | | |
| Sr | | Questionnaire | | Response | | Code | | | Skip | | |
| P29 | | Are some antimalarial drugs are banned in Myanmar? | | Yes  No  Don’t know | | 1  0  99 | | | 0,99🡪 P31 | | |
| P30 | | Which antimalarial drugs are banned in Myanmar?  ***Looking for either Generic name or Brand name. If “Don’t know”, enter “99” on the first line.*** | | [______________]  [______________]  [______________] | |  | | |  | | |
| P31 | | Have you heard/seen any messages or information about malaria in the past month? | | Yes  No | | 1  0 | | | 0🡪P34 | | |
| P32 | | Where did you see or hear these messages/information?  ***(Multiple response)*** | | TV  Radio  Billboard  Pamphlet  Newspapers/ Journals  Health Talk  Sales representative from AA pharma  PSI detailer  Others (Specify) (_____________________) | | **MR**  1  2  3  4  5  6  7  8  96 | | |  | | |
| P33 | | What type of malaria messages or information did you see or hear?  ***(Multiple response)*** | | Importance of giving full course of treatment | | **MR**  1 | | |  | | |
|  |  |  |  | Using the quality assured ACT | | 2 | | |  |  |  |
|  |  |  |  | Using diagnostic test | | 3 | | |  |  |  |
|  |  |  |  | Selling price | | 4 | | |  |  |  |
|  |  |  |  | Not to cut the strips | | 5 | | |  |  |  |
|  |  |  |  | AM monotherapy is dangerous | | 6 | | |  |  |  |
|  |  |  |  | Monotherapies are not recommended by WHO/NMCP | | 7 | | |  |  |  |
|  |  |  |  | Monotherapies are replaced by ACTs | | 8 | | |  |  |  |
|  |  |  |  | ACTs are recommended drug for malaria by WHO/NMCP | | 9 | | |  |  |  |
|  |  |  |  | ACTs are more effective | | 10 | | |  |  |  |
|  |  |  |  | ACTs have more attractive profit margin | | 11 | | |  |  |  |
|  |  |  |  | Quality seal logo on drug/facility | | 12 | | |  |  |  |
|  |  |  |  | Messages not related to ACT | | 13 | | |  |  |  |
|  |  |  |  | Do not remember | | 99 | | |  |  |  |
| P34 | | What does this logo on this drug mean?  ***(Podonma Show card)*** | | ***Quality malaria drug*** (correct answer)  Other responses (incorrect answer)  Do not know | | 1  2  99 | | |  | | |
| P35 | | Had someone from PSI ever visited you? | | Yes  No | | 1  0 | | | 0🡪P38 | | |
| P36 | | Had someone from PSI visited you in the last month? | | Yes  No | | 1  0 | | | 0🡪P38 | | |

| Sr | Questionnaire | Response | Code | | Skip | |
| --- | --- | --- | --- | --- | --- | --- |
| P37 | What kind of messages/information did he/she share with you?  ***(Multiple response)*** | Importance of giving full course of treatment | **MR**  1 | |  | |
|  |  | Using the quality assured ACT | 2 | |  |  |
|  |  | Using diagnostic test | 3 | |  |  |
|  |  | Selling price | 4 | |  |  |
|  |  | Not to cut the strips | 5 | |  |  |
|  |  | AM monotherapy is dangerous | 6 | |  |  |
|  |  | Monotherapies are not recommended by WHO/NMCP | 7 | |  |  |
|  |  | Monotherapies are replaced by ACTs | 8 | |  |  |
|  |  | ACTs are recommended drug for malaria by WHO/NMCP | 9 | |  |  |
|  |  | ACTs are more effective | 10 | |  |  |
|  |  | ACTs have more attractive profit margin | 11 | |  |  |
|  |  | Quality seal logo on drug/facility | 12 | |  |  |
|  |  | Messages not related to ACT | 13 | |  |  |
|  |  | Do not remember | 99 | |  |  |
| ***Section V: Cocktails*** | | | | | | |
| P38 | **(Do not ask this question to clinic)**  Does this outlet provide ‘***cocktail***, for the treatment of patients with uncomplicated malaria? | Yes  No | | 1  0 | | 0 🡪 P42 |
| P39 | **(Do not ask this question to clinic)**  Can you tell me, in this outlet are the ‘***cocktail***:  ***Interviewer read out responses. One response possible*** | Pre-made  Prepared at the time when customers come for treatment  Both | | 1  2  3 | | 2,3 🡪 P41 |
| P40 | **(Do not ask this question to clinic)**  Where do you obtain these ‘***cocktail***?  ***Interviewer read out responses. Multiple responses possible.*** | Pharmacy  Made in this outlet  Other ***(specify)*** [__________] | | **MR**  1  2  3 | |  |
| P41 | Please show me the ‘***cocktail*** you sell, or that you would prepare to sell, for adult man with symptoms of malaria and please tell me what are those?.  ***Interviewer to observe what the provider offers*** | [____________________________] [__] Tab  [____________________________] [__] Tab  [____________________________] [__] Tab  [____________________________] [__] Tab  [____________________________] [__] Tab  [____________________________] [__] Tab  [____________________________] [__] Tab  [____________________________] [__] Tab | | [_99 | |  |

| Sr | Questionnaire | Response | | Code | Skip | | |
| --- | --- | --- | --- | --- | --- | --- | --- |
| P42 | ***Do not ask the following 3 questions.***  ***Observe and circle the appropriate response in each case.***  Are medicines stored in a dry area? | Yes, stored in a dry area  No, not stored in a dry area  Did not observe medicines | 1  0  8 | |  | |  |
| P43 | Are medicines protected from direct sunlight? | Yes, protected from direct sunlight  No protections from direct sunlight  Did not observe medicines | 1  0  8 | |  | |  |
| P44 | Are medicines kept on the floor? | Yes, they are kept on the floor  No, not kept on the floor  Did not observe medicines | 1  0  8 | |  | |  |
| ***Section VI: Products tracking sheet*** | | | | | | | |
| 1 | Total number of Tablet, Suppository and Granule ***Products*** Audited | [___\|___\|___] |  | | |  | |
| 2 | Total number of Non-Tablet ***Products*** Audited | [___\|___\|___] |  | | |  | |
| 3 | Total number of RDT ***Products*** Audited | [___\|___\|___] |  | | |  | |

***Thank the provider for their participation.***

***Return to question C10 to record final status of interview and time of completion, then complete the section Ending the Interview.***
